# Mediterranean Diet for the Prevention of Gestational Diabetes in the Covid-19 Era: Implications of Il-6 In Diabesity

**DOI:** 10.3390/ijms22031213

**Published:** 2021-01-26

**Authors:** Anna Lucia Fedullo, Antonio Schiattarella, Maddalena Morlando, Anna Raguzzini, Elisabetta Toti, Pasquale De Franciscis, Ilaria Peluso

**Affiliations:** 1 Research Centre for Food and Nutrition, Council for Agricultural Research and Economics (CREA-AN), 00178 Rome, Italy; annalucia.fedullo@libero.it (A.L.F.); anna.raguzzini@crea.gov.it (A.R.); elisabetta.toti@crea.gov.it (E.T.); 2 Department of Woman, Child and General and Specialized Surgery, University of Campania Luigi Vanvitelli, 80138 Naples, Italy; aschiattarella@gmail.com (A.S.); madmorlando@gmail.com (M.M.); pasquale.defranciscis@unicampania.it (P.D.F.)

**Keywords:** cardio-diabesity, inflammation, oxidative stress, nutraceuticals, plant foods

## Abstract

The aim of this review is to highlight the influence of the Mediterranean Diet (MedDiet) on Gestational Diabetes Mellitus (GDM) and Gestational Weight Gain (GWG) during the COVID-19 pandemic era and the specific role of interleukin (IL)-6 in diabesity. It is known that diabetes, high body mass index, high glycated hemoglobin and raised serum IL-6 levels are predictive of poor outcomes in coronavirus disease 2019 (COVID-19). The immunopathological mechanisms of the severe acute respiratory syndrome coronavirus 2 (SARS-CoV-2) infection include rising levels of several cytokines and in particular IL-6. The latter is associated with hyperglycemia and insulin resistance and could be useful for predicting the development of GDM. Rich in omega-3 polyunsaturated fatty acids, vitamins, and minerals, MedDiet improves the immune system and could modulate IL-6, C reactive protein and Nuclear Factor (NF)-κB. Moreover, polyphenols could modulate microbiota composition, inhibit the NF-κB pathway, lower IL-6, and upregulate antioxidant enzymes. Finally, adhering to the MedDiet prior to and during pregnancy could have a protective effect, reducing GWG and the risk of GDM, as well as improving the immune response to viral infections such as COVID-19.

## 1. Introduction

The ongoing coronavirus disease 2019 (COVID-19) pneumonia pandemic, caused by the severe acute respiratory syndrome coronavirus 2 (SARS-CoV-2), is spreading worldwide [1]. In order to reduce the spread of the infection, the management of the pandemic requires several measures across all continents, such as local lockdowns. These lockdowns have potential long-term side effects on risk factors for cardiovascular diseases (CVD), such as unhealthy diet and physical inactivity [2]. Increased intakes of energy-dense processed foods (such as high fat meals and sweets) and/or reduced energy expenditure has been proven to lead to diabesity [3]. The latter, indicating the coexistence of type 2 diabetes (T2D) and obesity [4], can be associated with other CVD risk factors (such as dyslipidemia and hypertension) in the metabolic syndrome (MetSyn) [5]. T2D, obesity, MetSyn, and CVD are collectively known as cardiodiabesity [6,7].

**Figure 1:**
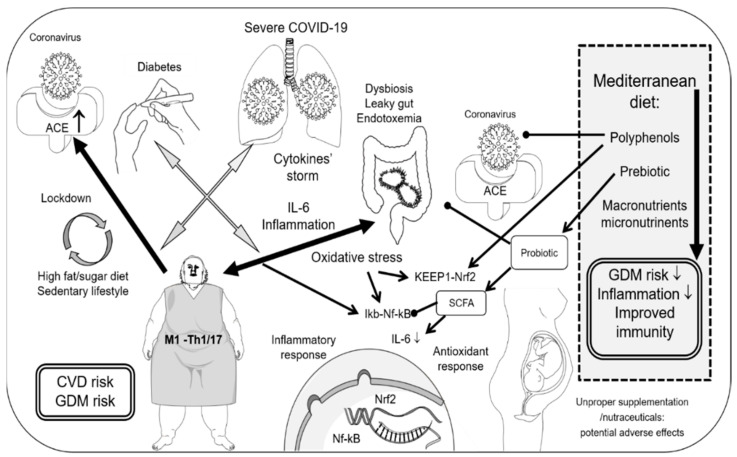
COVID-19, diabesity, and MedDiet interactions. ACE: Angiotensin-Converting Enzyme; COVID-19: Coronavirus Disease 2019, CVD: cardiovascular disease; GDM: Gestational Diabetes Mellitus; KEAP1: Kelch-Like ECH-Associated Protein 1; I-κB: Kappa b inhibitor; IL: interleukin; Nrf2: Nuclear Factor Erythroid-Derived 2-Like; SCFA: Short-Chain Fatty Acids.

There is a high prevalence of pre-existing comorbidities in patients with severe COVID-19 [8,9], with old age, smoking, hypertension, diabetes, and chronic obstructive pulmonary disease considered as risk factors for poor prognosis [10,11]. Individuals with diabetes—in particular those with higher body mass index (BMI) and with higher glycated hemoglobin (HbA1c)—are at higher risk of severe COVID-19 [11] (Figure 1). Patients with diabetes present an impairment in both innate and adaptive immunity that enhances their susceptibility to infections [12]. Moreover, the inflammatory response to viral infections is also boosted with a higher risk of cytokine storm [13,14]. Both malnutrition and over-nutrition are associated with impaired immune function and/or chronic low-grade inflammation [15,16].

Currently, the primary prevention of COVID-19 infection is based on the use of vaccines [17]. Monoclonal antibodies from El Lilly and Regeneron have been approved by the Food and Drug Administration (FDA) for treating COVID-19 patients [18], who also issued an emergency use authorization (EUA) for other drugs [19]. The available data suggest that the respiratory failure from acute respiratory distress syndrome (ARDS) is the leading cause of mortality. In a meta-analysis of 86 studies, severe COVID-19 was strongly associated with lymphocytopenia [20], hyperglycemia [21], ARDS [21], and high levels of interleukin (IL)-6 [20,21] and C-reactive protein (CRP) [20].

Despite the limited available data on COVID-19 in pregnant women, maternal mortality appears to be similar to non-pregnant women, while data on neonatal outcomes suggest an increased risk of complications [22,23,24]. Pregnancy can be associated with an impairment in glucose tolerance that could lead to the development of Gestational Diabetes Mellitus (GDM).

Non-pharmacological interventions in the management of diabesity include changes of lifestyle and dietary advice [25]. The effects of the ketogenic diet and of intermittent fasting on immunity and inflammation are controversial [16,26,27,28,29] due to the risk of ketoacidosis [29], which should be considered in women with GDM [30]. However, it is well known that the Mediterranean diet (MedDiet), rich in vegetables, legumes, nuts, cereals, and fish, is effective in preventing cardiodiabesity [6,7].

MedDiet has been suggested for health promotion in the COVID-19 era [31,32]. Furthermore, it has been proposed that a balanced diet might improve the immune response to SARS-CoV-2 infection [15,16]. Moreover, the adoption of a healthy lifestyle (including diet and physical exercise) can reduce the risk factors associated with the thrombotic complications of COVID-19 [33]. Obesity is related to the hyper-coagulopathy observed in severe COVID-19 [34], and pregnant women with COVID-19 showed increased D-dimer and fibrinogen levels [35]. However, it has been suggested that the fibrinolysis system of pregnancy may protect pregnant women with COVID-19 from the development of severe illness [35].

On the other hand, obesity, diabetes mellitus, and raised serum IL-6 levels were predictive of poor pregnancy outcomes in the course of COVID-19 infection [36]. Furthermore, increased levels of IL-6 were shown in the newborns of infected mothers [37,38]. IL-6 has a role in the development of insulin resistance and hyperglycemia [39,40,41,42]. Pregnant women affected by GDM have also shown elevated IL-6 concentrations, compared to controls [43,44]. Liu et al. analyzed 51 newborns from mothers with COVID-19 during the third trimester and found that an infant who developed necrotizing enterocolitis presented higher IL-6 levels, compared to the other children [38].

This review aims to discuss the potential of MedDiet and its components on GDM and Gestational Weight Gain (GWG), with particular reference to the adipokine IL-6.

## 2. Lockdown, Lifestyle and Diabesity: Implications for Pregnancy

Mediterranean lifestyle habits include diet, physical activity, and social interaction [45], and some Mediterranean pyramids include emotional balance [46,47] or psychological wellness [48], all factors that can be influenced by lockdown. A recent review revealed more frequent snack consumption and a rise in the intake of carbohydrates with a high glycemic index during the COVID-19 pandemic [49]. Stress and depression during quarantine have also led to unhealthy diets and reduced physical activity in some individuals [50]. The metabolic consequences of confinement include increases in insulin resistance, total body fat, abdominal fat, and inflammatory cytokines [10]. All of these factors have been strongly associated with the development of MetSyn, which in turn increases the risk of chronic diseases [10].

During the COVID-19 pandemic, the lockdown period has led some obese individuals to increases in body weight, mainly caused by decreased exercise, solitude, anxiety/depression, and increased consumption of snacks, unhealthy foods, and sweets [51].

Patients with T2D reported a high consumption of sugary food and snacks [52,53,54], as well as increased stress and reduced exercise [54]. Furthermore, associations were found between food cravings and snack consumption [52], increased snack consumption and/or decreased exercise levels and body weight gain [54], increased total diet intake and raised HbA1c concentrations [54]. A poor glycemic control was found in TD2 patients with an unhealthy diet and low physical activity [55]. T2D patients with mental stress also had unhealthy dietary habits [55].

During pregnancy, the psychological state is an important factor to consider because a high “Negative Affect Score” was found to reduce the favorable effects of a high “Mediterranean Diet Score” on the “Homeostasis Model Assessment of Insulin Resistance” (HOMA-IR) [56].

During lockdown, psychological stress was among the most common factor that worsened hyperglycemia, followed by changes in diet and exercise [57]. Furthermore, some patients required additional medications to control blood glucose levels during lockdown [57]. In women with GDM, the use of insulin therapy was significantly higher in 2020 (47.7%) compared with 2019 (36.2%) [58]. These results were explained by anxiety, dietary habits, and reduced physical activity [58]. In France, a higher rate of poor postprandial glycemic control was observed during the COVID-19 lockdown from 18 March 2020 to 7 May 2020 (13.7%), as compared with the same period during 2019 (8.7%) [58]. In Italy, it has been reported that diet therapy and/or insulin were sufficient to obtain a favorable maternal and fetal outcome in women with GDM and a concomitant SARS-Cov-2 infection [59]. In China, during the COVID-19 pandemic, a higher emotional eating score was associated with a significant excess of GWG [60]. After adjustments for exercise, the emotional eating score was also associated with a decreased consumption of fish and seafood [60].

In the “Croatian Islands Birth Cohort Study” adherence to the MedDiet ranged from low to moderate among pregnant women, with the highest adherence seen among women with healthier lifestyles [61]. Moreover, low adherence to a MedDiet pattern in healthy Spanish women before pregnancy was related to smoking habits and sedentary lifestyles [62]. However, a survey conducted in May 2020 reported that pregnant Spanish women decreased their physical activity and increased their sitting time after the confinement, whereas no differences were found in eating patterns [63]. Results from the “Australian Longitudinal Study on Women’s Health” reported that the MedDiet was inversely associated with the risk of developing gestational hypertension and pre-eclampsia [64]. Moreover, several follow-up studies in women with a history of GDM from the Nurses’ Health Study II cohort suggested that, in order to lower subsequent risk of developing hypertension [65] and T2D [66], it could be helpful to adhere to a healthy dietary pattern. The inverse association between T2D and healthy diets is partly mediated by BMI [66], and healthy diets are associated with lower weight gain regardless of other lifestyle factors [67].

## 3. Diabesity, Meta-Inflammation, and Il-6: The Role in Severe COVID-19

SARS-CoV-2 infiltrates human cells and binds to the angiotensin-converting enzyme 2 (ACE2) receptor, which is more expressed in individuals with diabetes and/or those in treatment with ACE inhibitors and angiotensin receptor blockers [68] (Figure 1). Dysregulation of the ACE2 pathway in the pancreas causes β-cell dysfunction and induces hyperglycemia [69]. The latter [28] and hyperinsulinemia [70] play a crucial role in thrombo-inflammation during COVID-19 infection. Countries with higher and lower death rates for COVID-19 appear to have high consumptions of fats and cereal, respectively [71]. Hyperglycemia and Reactive Oxygen Species (ROS)-induced oxidation of carbohydrates generate the adduct derivatives advanced glycation end products (AGE) [72]. The activation of the receptor for the advanced glycation end products (RAGE), which is also expressed in the lungs, produces a pro-inflammatory response via nuclear factor (NF)-κB, by increasing the NF-κBp65 expression and the degradation of IκB [73]. Accumulation of AGE could prime an exaggerated cytokine response to viral infection and ARDS [74]. It has also been suggested that SARS-CoV-2 could activate RAGE, a pattern recognition receptor that recognizes both pathogen-associated molecular patterns from microorganisms and danger-associated molecular patterns released by damaged cells [73,74]. Infection with SARS-CoV-2 also perturbs the renin–angiotensin system, favoring angiotensin II receptor 1 activation and potentially transactivating RAGE [74]. In overweight/obese individuals (BMI ≥ 27 kg/m^2^) the reduced ACE2 mRNA expression in subcutaneous adipose tissue was associated with an improvement of insulin sensitivity of skeletal muscle during weight reduction over 3 months [75]. Moreover, the small intestine expression of ACE2 receptors and respiratory virus infection is associated with dysbiosis of the gut microbiota [76] (Figure 1). Dysbiosis and endotoxemia have been implied in the observed increase of COVID-19 severity in obesity [77]. Metabolic endotoxemia could be induced by the direct diffusion of the Gram negative endotoxin lipopolysaccharide (LPS) from the intestinal lumen, due to intestinal paracellular permeability resulting from dysbiosis [78,79], or through the absorption by enterocytes during the secretion of chylomicrons in the postprandial state [80]. LPS activates “oxidative burst” in neutrophils and macrophages [81], leading to the formation of oxidized low-density lipoproteins and atherosclerotic plaques [82]. The overexpression and the activation (by LPS) of toll-like receptor (TLR)4 [78,82], as well as the ROS-mediated activation of p38 mitogen-activated protein kinase (MAPK) signaling and NF-κB [78,83], are involved in meta-inflammation. The latter [78] has been identified among mechanisms that link obesity, T2D, non-communicable diseases [79,83,84], and poor host response to viruses [14]. Meta-inflammation and chronic low-grade inflammation are observed in obese individuals, and characterized by high levels of circulating pro-inflammatory cytokines including IL-6 [78,79,85].

The mechanisms of SARS-CoV-2 infection include cytokine storm syndrome, an auto-amplifying cytokine production that occurs due to an unregulated host immune response, and involves IL-6. The IL-6 receptor antagonist tocilizumab has been used in some cases of severe COVID-19 [86,87,88,89,90]. However, the benefits of tocilizumab for the treatment of COVID-19 are uncertain, [91,92,93,94,95,96,97,98,99,100] and the Italian Medicines Agency (AIFA) announced on June 18, 2020 that tocilizumab (Actemra) did not improve COVID-19 outcomes [91,92,93]. However, a recent meta-analysis reported a reduced mortality prevalence in patients treated with tocilizumab [97]. Sarilumab is another IL-6 receptor antagonist under investigation in clinical trials [91,98,99]. In addition to IL-6 receptor targeting, clinical trials with anti-IL-6 treatments (clazakizumab and siltuximabin) are ongoing [99]. However, more studies are needed in order to assess the safety and efficacy of IL-6 signal inhibitors [100].

Only two case reports demonstrated successful tocilizumab therapy in pregnant women with TD2 [101] or GDM [102], however one of these observed transaminitis and hyper-triglyceridemia after treatment [102]. Although pregnancy is not a significant risk factor for severe COVID-19, obesity in pregnant women represents a key co-morbidity and high circulating levels of leptin have been associated with an increased mortality in patients with ARDS [103]. Moreover, the increased levels of leptin are pro-inflammatory [93] and could induce macrosomia in the fetus [104].

The expansion of adipose tissue promotes macrophage infiltration and the production of leptin and other inflammatory adipokines, including Tumor Necrosis Factor alpha (TNF)-α and IL-6 [85]. IL-6 and CRP are associated with hyperglycemia and insulin resistance [78]. IL-6 has also been suggested as a marker able to predict T2D development [78]. IL-6 is a critical signaling molecule released from fat cells and belongs to obesity-related cytokines [105,106].

The causes of GDM remain uncertain, but recent literature has hypothesized the pivotal role of the immune system in inception and development of this disease. Other components have been involved in the pathogenesis of GDM such as oxidative stress and obesity. Several studies also underlined that an imbalance between T helper (Th)1 and Th2 cells has a critical role in cytokine production in women affected by GDM [107]. During normal pregnancy, T helper cells showed a pro-inflammatory Th1 profile, by mediating the production of interleukin IL-2, interferon (IFN)-γ, and TNF-α. Higher levels of these molecules have been found in patients affected by GDM compared to controls [108]. On the other hand, the Th2 profile is characterized by an anti-inflammatory response through the production of several interleukins such as IL-10, IL-4, IL-5, and IL-13. [109]. Elevated concentrations of IL-6 and CRP might predict the onset of GDM [110].

It is well known that fat distribution has a role in obesity-induced T2D [111]. Peripheral obesity is associated with “alternatively activated” M2 macrophages, Th2, and regulatory T cells [111]. The latter produce IL-10, which could inhibit the ability of TNF-α to downregulate the Glucose Transporter(GLUT)-4 expression and to impair the insulin action in adipocytes [112]. However, individuals with central obesity are characterized by infiltration of proinflammatory Th1, Th17, and “classically activated” M1 macrophages [111] (Figure 1). In adipose-tissue biopsies of overweight/obese individuals, proinflammatory Th1 and Th17 were more frequent in visceral adipose tissue (VAT) than in subcutaneous adipose tissue (SAT) [113]. Recent studies found that pregnant women had higher total lymphocyte counts in VAT compared to SAT regardless of GDM [114] and that higher VAT better predicts GDM than pre-pregnancy BMI [115]. Infiltration of proinflammatory Th cells into VAT is recognized as one of the primary events in obesity-induced chronic inflammation, and diet-induced obesity promotes the expression of T-cell co-stimulatory molecules on the surface of adipose tissue macrophages and of VAT resident dendritic cells [112]. Dendritic cells are the major contributors to the induction of T17 cells in adipose tissue possibly via expressing high levels of IL-6, transforming growth factor-β, and IL-23 [112]. The role of IL-6 is controversial because it is an inflammatory cytokine involved in Th17 differentiation and its plasma concentration correlated with Th1 in SAT from adipose-tissue biopsies of overweight/obese individuals [113], but IL-6 release from exercising muscle has been suggested to inhibit the LPS-induced TNF-α production [116], limiting inflammation during endotoxemia [117]. Moreover, IL-6 induced expression of the receptor for IL-4 and increased the response to IL-4 in macrophages and their alternative activation [117,118]. Obesity is accompanied by a switch in macrophage activation from the M2 macrophages to the proinflammatory M1 macrophages [112], and the hyperactivation of M1 macrophages is linked to aerobic glycolysis and hyperglycemia [28]. Data from animal models suggest that pregnancy is associated with an alternatively activated phenotype of alveolar macrophages before infection, which may contribute to the increased inflammatory response to influenza virus infection and to heightened disease severity [119]. Furthermore, the polarization of macrophages has a role in pre-eclampsia, recurrent spontaneous abortion, infertility, intrauterine growth restriction, and preterm labor [120]. In a case-control study, peripheral blood monocytes showed that pro-inflammatory M1-like phenotype and M1 macrophage infiltration was increased in the placenta of women with pre-eclampsia compared with controls, and pro-inflammatory factors—including IL-6—were significantly increased in the serum and placenta of women with pre-eclampsia [121]. Data from animal models suggested that the adipose tissue M1 or M2 phenotypes under postnatal high fat diet in offspring was affected by maternal undernutrition due to protein restriction [122]. Moreover, within adipose tissue macrophages there was an increase of IL-6 gene expression [122].

The impact of different dietary approaches on metabolism, inflammation, and long-term outcomes has been extensively studied. In a systematic review and meta-analysis of 74 trials evaluating the effects of high- versus low-protein diets, a higher reduction of BMI, waist circumference, and fasting insulin were observed with the high-protein diet, however the effects on fasting glucose and HbA1c were not significant between the two diets and adverse gastrointestinal events were also reported with the high-protein diet [123]. In this context, it is known that diet rapidly alters microbial community [124] and a position paper suggested caution regarding the use of high-protein diets in the long-term, due to the effect on microbiota and gene expression in the gut [125]. Despite the potential advantages for the metabolism of anti-inflammatory M2 macrophages by the reduction of glucose availability for activated M1 macrophages through an eucaloric ketogenic diet [28], Tagliabue et al. reported that prolonging the ketogenic diet for 3 months increased *Desulfovibrio* spp., which is involved in the exacerbation of the inflammatory condition of the gut mucosa associated with the consumption of fats of animal origin [126]. Furthermore, in diabetic patients, restriction of carbohydrates to below 26% of total energy, compared to a diet with carbohydrates at more than 45% total energy produced greater reductions in HbA1c at 3 and 6 months, but no significant difference at 12 or 24;months were found [127]. A consensus statement from the Italian Society of Endocrinology [128] concluded that concern still exists about potential risks of very-low-calorie ketogenic diets in the long-term.

MedDiet—as well as low-carbohydrate, low-glycemic index, and high-protein diets—decreased HbA1c compared with their control diets, but the greater effect was observed with the MedDiet as it also reduced body mass significantly [129]. Data from meta-analysis in individuals with T2D and/or prediabetes reported that MedDiet reduced fasting glucose and body mass compared to other diets, including the diet suggested by the American Diabetes Association and the European Association for the Study of Diabetes (50–55% carbohydrates with mixed glycemic index, 30% fat and 15–20% protein) [130].

## 4. Mediterranean Diet for Prevention of Obesity and Gestational Diabetes

The Western diet, characterized by a high consumption of sugar and saturated fats contributes to the predominance of obesity and T2D, activates the innate immune system, and impairs adaptive immunity. These lead to chronic inflammation and an impaired host defense against viruses, which could place people in this category at an increased risk for severe COVID-19 [131].

The role of an elevated BMI in the spreading of GDM has been widely analyzed by previous studies [132,133] and it is known that it highly impacts the immune system and the related increase of circulating inflammatory cytokines during pregnancy [134]. However, it has been speculated that cytokine production could be enhanced by other sites besides adipose tissue such as immune cells in placenta [135].

An observational study on pregnancy of women comparing the western dietary pattern, a traditional dietary pattern, and a healthy dietary pattern (high in green, leafy and colored vegetables) demonstrated a borderline significant inverse association between high adherence to a healthy dietary pattern and the chance of pre-eclampsia [136]. Both Dietary Approaches to Stop Hypertension (DASH—including fruits, veggies, whole grains, and low-fat dairy) and MedDiet were related to lower fasting blood glucose, HbA1c, and serum triacylglycerol levels. High-density lipoprotein cholesterol was higher for those following DASH while total serum cholesterol was lower for those in the MedDiet. Participants that had high adherence to the MedDiet had 80% lower risk for GDM, while greater adherence to the DASH pattern was associated with 71% reduced risk for GDM [137]. Lower risks or odds of GDM were associated with higher physical activity levels before or in early pregnancy and to MedDiet/DASH [138]. These results strengthen the importance of dietary advices in pre-gravid women [139]. As usual, the target weight will differ according to the physiological and/or pathological state of women, and consequently the energy intake should be personalized [140].

Several studies described an inverse association among GWG [141] and GDM [142] related to MedDiet, whereas adequacy of vitamins (B9, D, and E) and dietary fibre were related to the adherence to MedDiet during pregnancy [143]. GWG seems to be related to the intakes of added sugar, but not to saturated fat or protein. In order to limit GWG, it was found to be relevant to reduce foods that contain added sugar such as snacks, sweets, and soft drinks [144]. However, an elevated risk of deflection from recommended GWG was observed in women with low MedDiet adherence [145]. A high baseline adherence to the MedDiet was linked to a lower weight gain during pregnancy and thus may protect against obesity and becoming overweight [146]. Following a MedDiet pre-pregnancy seems to be effective in preventing GDM and other maternofetal outcomes [147]. Compliance with MedDiet before the 12th gestational week was associated with a reduction of GDM [148] and with health benefits for the offspring, particularly in women with a pre-gestational BMI lower than 25 kg/m^2^ and normal glucose tolerance [149]. It was proven that also a late first-trimester (>12 gestational weeks) degree of adherence to a MedDiet pattern seems to positively impact on a composite materno-fetal outcome (CMFCs) such as perineal trauma, emergency C-section, pre-eclampsia, pregnancy-induced hypertension, prematurity, and large or small-for-gestational-age (SGA). The risk of GDM, CMFCs, prematurity, and SGA newborns decreased linearly with high, moderate, and low adherence to MedDiet [150,151,152].

A critical role in the progression of GDM could be proven by the inflammasome activation. This is mediated by caspase-1 and increases IL-1β concentration, a pro-inflammatory cytokine that impacts on insulin resistance. Higher levels of these molecules have been found in women affected by GDM [153]. Moreover, women with GDM had higher HbA1c levels at 24–28 gestational weeks compared to controls while the values were similar at 36–38 gestational weeks. Similarly, fasting serum insulin and HOMA-IR were higher in women with GDM at 24–28 gestational weeks but became similar at 36–38 gestational weeks [154]. Adherence to a MedDiet nutritional approach is related to a lower incidence of GDM and a better glucose tolerance (even in women without GDM) [152] and to a decreased risk of MetSyn [155]. The “Seguimiento Universidad de Navarra” project assessed the association of total, processed, and unprocessed red meat and iron intake with the risk of developing GDM in pregnant women [156]. It was found that a higher risk of GDM was significantly associated with total meat consumption, especially for red meat and processed meat. Non-heme and total iron intake, including supplements, are not associated with GDM. On the contrary, heme-iron intake was directly associated with GDM, but the statistical significance was lost when adjusted for red meat consumption [156]. Furthermore, a consumption of more than two sugar-sweetened soft drinks per week was an independent risk factor for GDM [157].

Transmission of obesity across generations is a reason of concern. High offspring adiposity at birth and overweight status in childhood may be associated with pre-pregnancy overweight/obesity and with an excessive GWG, respectively [158]. High birth weight, which is linked to subsequent MetSyn, seems to be linked with a high intake of carbohydrate during pregnancy combined with impaired glucose tolerance [159]. There is strong evidence that epigenetic changes during fetal development play a fundamental role in the development of MetSyn. These changes are induced by maternal nutrition, among different factors, affecting the intrauterine environment. Likewise, the MedDiet could have a similar action during pregnancy, protecting the fetus against the development of MetSyn throughout life [160]. In obese women, even those without GDM but with impaired glucose tolerance, a lower carbohydrate intake at moderate levels in late gestation is associated with a lower fat mass in their offspring at birth [160].

Growing evidence advises on the beneficial effect of the MedDiet for women of reproductive age and during pregnancy on children’s health against prematurity, fetal growth, neural tube defects and other congenital pathologies, as well as against asthma and allergies, body weight, and metabolic markers [161,162]. In fact, children born from mothers that followed a MedDiet during pregnancy are protected against cardiometabolic risk such as blood pressure, and blood levels of lipids, CRP, and adipokines [163]. Therefore, MedDiet lifestyles should be incorporated into strategies for prevention and treatment of overweight/obesity especially in women of childbearing age [158]. Compared to controls, women with GDM had higher rates of insufficient weight gain, SGA, and neonatal intensive care unit admission, while the rates of macrosomia, large for gestational age, pregnancy-induced hypertensive disorders, prematurity and cesarean sections were similar [154]. In addition, maternal regimen during pregnancy might also influence the development of childhood allergic disorders. An increased risk of wheeze in the first year of life was associated with high meat intake and processed meat intake during pregnancy, while a high intake of dairy foods was significantly associated with a decreased risk [164]. Finally, high adherence to the MedDiet during pregnancy seems to be associated with a reduced incidence of allergic sensitization, allergic rhinitis, and wheeze in the first year of life [165,166].

## 5. Mechanisms of the Mediterranean Diet Components

It is well known that omega-3 polyunsaturated fatty acids (omega-3 PUFA), vitamins (folate, A, B6, B12, C, D, and E) and minerals (copper, iron, magnesium, selenium, and zinc), improve the immune system [167,168,169,170,171,172,173]. Although supplementations with omega-3 PUFA and/or micronutrients have been suggested to improve clinical outcomes of patients with ARDS [174,175,176,177] and to improve public health [167,177], it has been pointed out that only individuals at high-risk of specific nutrient deficiencies could benefit from supplementation, whereas the supplementation of a single nutrient is not promising in the general population [178]. Moreover, it has been recently found that zinc supplementation in pregnant women decreased hemoglobin concentration [179]. On the other hand, it has been suggested that vitamin D could modulate IL-6 concentrations [180], whereas an appropriate magnesium status activates the functionality of vitamin D and reduces IL-6, CRP, and NF-κB [181]. In pregnant women, supplementation with vitamin D did not improve their IL-6 serum but it was higher in the 1000 IU/d (25.9 ± 32.0 ng/L) than in 2000-IU/d group (4.6 ± 1.4 ng/L) [182].

Vitamin D has been identified as a potential candidate in the prevention of GDM [183]. Data from a recent meta-analysis reported that the combination of exercise and diet or supplementation with vitamin D reduced GDM risk [184]. In consideration of the lockdown measures, the reduced exposure to sunlight and therefore the consequent reduction of endogenous vitamin D production, could represent a critical point on the development of this pathology. The supplementation with docosahexaenoic acid (DHA) and multiple micronutrients during the second and third trimesters of pregnancy improved women’s DHA and vitamin D status compared to controls, without affecting significantly the glutathione status and 8-isoprostane [185]. Moreover, in a recent meta-analysis omega-3 PUFA did not reduce the risk of GDM, whereas more studies are needed to establish the effect of vitamins, minerals, or probiotics [184].

MedDiet can be considered a natural supplement that includes fibre, omega-3 PUFA, and polyphenols, with the gut microbiota appearing to be the main target and player in the interactions occurring between omega-3 PUFA, polyphenols, probiotics, and prebiotics [186,187].

Some phenolic compounds are able to induce insulin secretion (apigenin, cyaniding, delphinidin, catechins, and quercetin) [81] and increase the GLUT4 insulin-mediated glucose uptake in adipocytes or skeletal muscle cells (catechins, procyanidins, and phenols of extra virgin olive oil (EVOO)) [188,189]. Furthermore, plant-foods’ bioactive compounds inhibit the GLUT2 and the sodium-dependent glucose transporter (catechins and quercetin), as well as the activity of α-glucosidase (anthocyanins, catechins, flavanones, flavones, flavanols, and isoflavones) or α-amylase (quercetin, luteolin, and myricetin) [81,188,189]. These mechanisms are among the major strategies for reducing the risk of diabetes including slowing (increase fibre consumption) or reducing (amylase and glucosidase inhibition) carbohydrate absorption [190]. However, diarrhea and flatulence are the most frequent side-effects to acarbose, due to its inhibition of starch digestion [191] and fibre can lead to similar gastrointestinal problems [190]. It has been suggested that when the COVID-19 infection involves the intestine of obese individuals, dysbiosis could contribute to the cytokine storm [192]. Dietary fats induce dysbiosis and may increase intestinal permeability, inducing endotoxemia and chronic low-grade inflammation [192], whereas polyphenols modulate microbiota composition [186]. Although antiviral properties against SARS-CoV-2 (related to virus entry into the cells) have been reported in vitro for some bioactive phytochemicals (Figure 1), including the hesperidin [193,194], quercetin [194,195], kaempferol [194], catechins [194,196], baicalin [197], curcumin [194] and resveratrol [194], their availability in humans is low [198]. Only 5–10% of dietary polyphenols can be absorbed and they undergo to microbial metabolism in the colon [188]. However, it has been proposed that the products of microbial fermentation of dietary polyphenols could be responsible for antioxidant and anti-inflammatory activities and that polyphenols enrich the gut with beneficial bacteria [188] (Figure 1). In this context, it has been recently suggested that tailored nutrition and supplementation, known to improve the intestinal microbiota and its immune function, might help to minimize the impact of the disease at least on people at higher risk from COVID-19 [199,200,201,202]. Probiotics, prebiotics, dietary fibre, and their symbiotic combinations improve health through the production of short-chain fatty acids (SCFA) that modulate immune functions [76,202,203], inhibiting the NF-kB pathway and lowering IL-6 [204,205] (Figure 1). Moreover, it has been suggested that β-glucan from the baker’s yeast *Saccharomyces cerevisiae*, with a diet rich in vitamins C and D, can improve microbiota and immune response to respiratory virus infections, and reduce inflammation and coagulation abnormalities [206]. Furthermore, low-purine diets and *Lactobacillus gasseri* reduce intestinal purine absorption and have been suggested for improving the immune system and weakening viral replication in individuals with hyperuricemia [207]. Although uric acid is the major antioxidant in biological fluids [72,208], at concentrations above the saturation level it can be a dangerous signal for the immune system [82]. In order to decrease uric acid it is important to reduce the consumption of sardines, liver and kidneys [207], as well as fruit juices (containing fructose) [209] and alcoholic beverages [207,210]. Although the green tea epigallocatechin gallate can reduce uric acid [211,212], it can also activate the nuclear factor erythroid-derived- 2-like-2 (Nrf2)/ antioxidant responsive element (ARE) signaling by pro-oxidant mechanisms [213]. Moreover, it has been pointed out that high consumption of green tea is associated with low serum folate levels during pregnancy and an increased risk of neural tube defects [198] and case reports of severe anemia after consumption of large amounts of tea have been described [214,215]. In agreement, *Camellia sinensis* products are among the herbal extracts used to enhance immune system function for which the safety should be determined in pregnancy [216]. It has recently been pointed out that pregnant women are under-represented in clinical studies, and there is a need for more evidence with respect to drug and nutraceuticals safety and pharmacokinetics [217]. Although, in animal models, some bioactive phytochemicals—including resveratrol—had positive effects on embryo/fetal development and maternal health, however others—such as epicatechin gallate and curcumin—had adverse effects [198] and caution in the consumption of soy food and phytoestrogens during pregnancy has been suggested [198].

The mechanism suggested for Nrf2 and/or NF-κB modulation by polyphenols is the interaction of electrophiles with cysteine residues of Kelch-like ECH-associated protein 1 (KEAP1), I-κB, and/or I-kappa kinases [218] (Figure 1). Preclinical evidence indicates that lactic acid bacteria can modulate both Nrf2 and NF-κB pathways, and that sulforaphane, produced by the gut microbiota from the glucoraphanin contained in cruciferous (Brassica) vegetables, activates Nrf2 [219]. Moreover, it has been proposed that fermented cabbage may induce the Nrf2-mediated antioxidant response, and consequently reduced IL-6 [219]. The ROS-mediated activation of Nrf2, with resulting upregulation of antioxidant enzymes is a common mechanism of plant food polyphenols [78,189]. Polyphenol-rich foods and beverages include fruit, vegetables, cereals, EVOO, nuts, herbs, spices, coffee, chocolate, tea, and red wine [198,220]. Therefore, Nrf2 is among the factors involved in the hormesis proposed to explain the anti-inflammatory and antioxidant effects of the MedDiet [78,189,221]. In particular, some dietary phytochemicals—including phenolic antioxidants, glucosinolates, and resveratrol—act in a hormetic-like manner through the modulation of stress-response pathways and are called hormetins [221]. Nrf2 activity in response to ROS and xenobiotics is regulated by the thiol-rich protein KEAP1 [72,219] (Figure 1). The induction of the ARE and of the ROS-mediated pathway by Nrf2 reduces the activity of NF-κB [219]. NF-κB is localized in the cytoplasm associated with the I-κB inhibitor (Figure 1), and under the effect of inflammatory cytokines, including IL-6, I-κB is degraded and NF-κB translocates into the nucleus and induces the expression of cytokines, inducible nitric oxide synthase and cyclooxygenase 2 [222]. Inflammatory cytokines and ROS activate the MAPK [222], involved in the concerted modulation of redox regulated Nrf2 and NF-*κ*B gene expression in inflammation and carcinogenesis [223]. Studies in vitro or in animal models reported that resveratrol, naringin and hesperidin reduced IL-6 and/or the activation of NF-*κ*B and p38 MAPK pathway [220]. IL-6 and p38-MAPK are also involved in the over-expression of p-glycoprotein, inducing chemoresistance [223]. In this context, it has been suggested that MAPK could be a target of EVOO compounds, including squalene acting as “natural delivery system” of bioactive phytochemicals [223]. Moreover, EVOO has a role in the anti-inflammatory effects of the MedDiet [224], also reducing IL-6 [225]. Both polyphenols and omega-3 PUFA inhibit NF-*κ*B, MAPK, and/or TLR4 pathways [220,222]. However, in a meta-analysis of intervention studies, no significant difference was detected between flavonoid ingestion and control for the differences in plasma IL-6 concentration [226]. Omega-3 PUFA reduced IL-6 in preclinical studies and in elderly and obese individuals [222] but did not affect IL-6 levels in healthy individuals [222]. Omega-3 PUFA have been suggested for ameliorating the lung damage that occurs during COVID-19 [227]. However, omega-3 PUFA could make membranes more susceptible to ROS [228] and it has been suggested that vitamin E protects them and the immune cells from oxidation [205]. Therefore, MedDiet rather than single supplementation could be useful for health. Accordingly, data from a meta-analysis reported that while dietary patterns rich in polyphenols reduced the risk of GDM despite polyphenol-rich food groups—including polyphenol-rich fruit (berry, apple, and other pome fruit) and non-alcoholic beverage consumption (tea, cocoa, coffee, orange, and apple juices)—whole grains and legumes did not [229]. Furthermore, the consumption of potatoes or drupe (cherry, nectarine and plum) could increase the risk of GDM [229]. On the contrary all the included studies on MedDiet reported a significantly lower risk of GDM among women with the highest score of adherence [229].

## 6. Conclusions

The COVID-19 pandemic generates a great interest in dietary advice, supplements, nutraceuticals, and complementary/alternative medicine (CAM) in order to improve immune function [170,230,231,232]. However, there is no evidence for their efficacy in COVID-19 [170,230,231] and further studies are needed before giving public messages [170,231,232] in order to avoid potential adverse effects of CAM [232]. It has been reported that Chinese migrant women with GDM were more prone to supplementation use compared with Australian-born white women [233]. On the other hand, a cross-sectional study reported that many pregnant US women were at risk of excessive intake of iron and folic acid, despite others not meeting the recommendations for micronutrients even with the use of supplements [234]. In this context, the position of the Academy of Nutrition and Dietetics is that although pregnant women are among the individuals at particular risk for inadequate dietary intakes of micronutrients, the routine and indiscriminate use of supplements for the prevention of chronic disease is not recommended [235]. More studies are needed in order to evaluate the use of complementary medicine during pregnancy for treating pregnancy-induced nausea [236] and mood status [237]. The former can be due to ketosis [30] and the latter can be increased by lockdown [57,58,60,238].

Psychological stress was among the most common factors to worsen hyperglycemia, followed by changes in diet and exercise, and some patients required additional medications for the control of blood glucose during lockdown [57]. Therefore, the previously suggested multi-level approaches to assist individuals in adapting their health behaviors to prevent both chronic and infectious diseases [239] should be applied to pregnant women.

From the reviewed evidence in this work, it can be concluded that adhering to the MedDiet prior to and during pregnancy could be the better approach to reduce GDM risk and improve immune function. An early MedDiet plan is useful for both mother and offspring in order to reduce composite materno-fetal adverse outcomes [240]. Moreover, a lifestyle that includes physical exercise beyond diet could also promote a better control of weight gain and reduce waist circumference [241]. Therefore, the MedDiet could be considered a useful dietary option during pregnancy.

## Data Availability

No new data were created or analyzed in this study. Data sharing is not applicable to this article.

## Abbreviations

ACE	Angiotensin-Converting Enzyme
AGE	Advanced Glycation End Products
ARDS	Acute Respiratory Distress Syndrome
ARE	Antioxidant Responsive Element
BMI	Body Mass Index
CMFCs	Composite of Materno-Fetal Outcomes
CAM	Complementary/Alternative Medicine
COVID-19	Coronavirus Disease 2019
CRP	C-Reactive Protein
CVD	CardioVascular Disease
DASH	Dietary Approaches to Stop Hypertension
DHA	Docosahexaenoic Acid
EVOO	Extra Virgin Olive Oil
GDM	Gestational Diabetes Mellitus
GLUT	Glucose Transporter
GWG	Gestational Weight Gain
HbA1c	Glycated Hemoglobin
HOMA-IR	Homeostasis Model Assessment of Insulin Resistance
IFN	Interferon
I-κB	Kappa b inhibitor
IL	Interleukin
KEAP1	Kelch-Like ECH-Associated Protein 1
LPS	Lipopolysaccharide
MAPK	Mitogen-Activated Protein Kinases
MedDiet	Mediterranean Diet
MetSyn	Metabolic Syndrome
NF-κ	Nuclear Factor Kappa
Nrf2	Nuclear Factor Erythroid-Derived 2-Like
omega-3 PUFA	Omega-3 Polyunsaturated Fatty Acids
RAGE	Receptor for Advanced Glycation End-Products
ROS	Reactive Oxygen Species
SARS-CoV-2	Severe Acute Respiratory Syndrome Coronavirus 2
SAT	Subcutaneous Adipose Tissue
SCFA	Short-Chain Fatty Acids
SGA	Small-For-Gestational-Age
T2D	Type 2 Diabetes
Th	T Helper
TLR	Toll Like Receptor
TNF	Tumor Necrosis Factor
VAT	Visceral Adipose Tissue
